# Smoking, an additional risk factor in elder women with primary open-angle glaucoma

**Published:** 2009-12-31

**Authors:** Vicente Zanon-Moreno, Jose J. Garcia-Medina, Vicente Zanon-Viguer, Maria A. Moreno-Nadal, Maria D. Pinazo-Duran

**Affiliations:** 1 Biomedical Research Center, Dr. Peset University Hospital, Valencia, Spain; 2 Ophthalmology Research Unit ‘Santiago Grisolia’, Valencia, Spain; 3 Department of Ophthalmology, La Inmaculada Hospital, Huercal Overa, Almeria, Spain; 4 Department of Preventive Medicine & Public Health, Dr. Peset University Hospital, Valencia, Spain; 5 Department of Ophthalmology, Dr. Peset University Hospital, Valencia, Spain

## Abstract

**Purpose:**

Smoking is a serious public health problem worldwide. Some authors refer to it as the “silent epidemic of the 20th century.” It constitutes an important risk factor for ocular pathologies such as age-related macular degeneration (ARMD), diabetic retinopathy, and neuropathy because the toxic effects of tobacco play a key role in the deterioration of eye tissue. Damage to trabecular meshwork cells (TMC) and retinal ganglion cells (RGC), involving inflammation and apoptosis mechanisms, has been proved in glaucoma. The aim of this study was to determine whether smoking influences the progression of primary open angle glaucoma (POAG) in women.

**Methods:**

This experimental study involved a sampling of consecutive cases of smokers, ex-smokers and non-smokers women with POAG. One hundred and twenty women with POAG, aged 40–90 years, were enrolled (40 smokers, 40 ex-smokers and 40 non-smokers). Samples of aqueous humor (AH) and plasma from each subject were obtained at the beginning of the surgical procedures. Both inflammation and apoptosis processes in the subjects were studied by means of enzyme immunoassay and western blot procedures respectively. We analyzed the interleukin-6 (IL-6) levels as an inflammation marker and the expression of caspase-3 and poly (ADP-ribose) polymerase 1 (PARP-1) as apoptosis markers.

**Results:**

IL-6, caspase-3, and PARP-1 levels were significantly higher in the smoker women who smoked than in the ex-smoker and non-smoker glaucomatous groups of the same gender (p<0,05).

**Conclusions:**

Inflammation and apoptosis marker levels increase with smoking in the aqueous humor and plasma samples of POAG women. Smoking could be an important additional risk factor for glaucoma progression in elderly women.

## Introduction

Glaucoma is a syndrome characterized by high intraocular pressure (IOP), an alteration of the optic nerve head and a loss of visual field. When the loss of optic nerve tissue is significant, patients suffer deterioration in vision. Before this loss is quantifiable, patients may lose a significant amount of optic nerve tissue, making the early diagnosis of this disease critical [1]. Glaucoma is the major cause of irreversible blindness worldwide. There are 66.8 million people in the world who have glaucoma, with 6.7 million of them having bilateral blindness [2,3]. Risk factors for glaucoma include age, race, diabetes, myopia, hypertension, cardiovascular diseases, and a family history of glaucoma [4,5].

The genetic component of this disease is now undeniable. Several research groups have studied the relationship of certain genes to glaucoma and certain mutations in some of these genes have been associated with an increased risk of developing glaucomatous disease [6,7]. But not only are genetic factors key to the development and evolution of glaucomatous optic neuropathy, but so are environmental factors.

Snuff usage, according to World Health Organization data, is the leading cause of illness, disability, and premature death in the world, which means that smoking is a serious global public health concern. Of the approximately 1.3 billion smokers in the world, some 6 million die annually because of the effects of using tobacco [8]. Nowadays, smoking is considered a chronic addictive disease that causes problems not only for the people who do it, but also for those who live with them.

Smoking is associated with many diseases, including allergies, hypertension, heart disease, and lung cancer [9,10]. It is also related to eye diseases, mainly with optic neuropathies and age-related macular degeneration [11,12]. Smoking causes high oxidative stress because tobacco smoke contains oxidizing agents that produce free radicals [13]. A free radical is a molecule with one or more non-paired electrons in its outer orbit, which makes it very unstable and active. So the free radical will steal an electron from another molecule and, in this way, create another free radical. This produces a chain reaction which, if not stopped, will lead to cell damage and even cell death by apoptosis [14].

It is known that a significant loss of trabecular meshwork cells (TMC) occurs in patients with glaucoma in an age-related fashion [15]. Experimental studies in human [16] and animal models [17] have shown that TMC and  retinal ganglion cells (RGC) die by apoptosis in glaucoma. However, the switch-window in which cells start failing in their normal functions and appear to undergo apoptotic cell death in both the anterior and posterior eye segments in glaucoma remains unknown. Understanding the molecular processes involved in apoptosis regulation is a pivotal aspect for developing proper anti-glaucoma therapies.

It has been recently discovered a caspase-independent pathway of apoptosis that is driven by the nuclear enzyme poly ADP ribose polymerase 1 (PARP-1) [18]. The PARP enzymes constitute a family of cell signaling enzymes, also known as the poly (ADP-ribose) synthetases and transferases, which catalyze the poly (ADP-ribosylation) of DNA-binding proteins. Of the PARP family, PARP-1 was the first to be characterized and up to now is the best known member. In seriously damaged cells, when apoptosis is occurring, PARP-1 is the target of caspase-3, which proteolizes the protein and divides it into two fragments. One (the 85 kDa fragment) contains the COOH-terminal domain (catalytic domain), and the other (24 kDa) contains the NH_2_-terminal domain (DNA union domain). This proteolysis mechanism prevents DNA repair and allows the cell to initiate apoptosis [19]. In this context, overactivation of PARP-1 has been demonstrated to be closely related to the pathogenic mechanisms of various diseases, such as myocardial infarction [20], diabetes mellitus [21], neurodegenerative disorders [22], and inflammatory processes [23]. It has been shown that cigarette smoke can induce inflammatory responses [24-26], and researchers have been detected changes in the levels of several markers of inflammation (e.g. IL-6 or IL-8), probably due to the effects of smoke [27-29].

Some cytokines have also been studied in relation to glaucoma [30,31] (e.g., IL-6) because inflammatory processes have been associated with this optic neuropathy. Not only has IL-6 been associated with inflammatory responses, but it has also been linked to apoptotic processes [32-35]. Tobacco has also been associated with apoptotic processes because of the toxic substances it contains [36,37], and it is known that TMC and RGC die by apoptosis in glaucoma [38,39].

Taking all this into account, the aim of this study was to analyze possible changes in the expression of pro-apoptotic proteins (PARP1, caspase 3) and the levels of inflammatory markers (IL-6) in aqueous humor and plasma of subjects with POAG.

## Methods

The Doctor Peset University Hospital Review Boards and Committees approved the protocols, which adhered to the Helsinki guidelines for human research. An informed consent agreement was signed by all study participants.

### Subjects

Patients were examined by experienced ophthalmologists in the corresponding departments of the study centers. A systematized ocular examination including visual acuity, IOP, biomicroscopy, ocular fundus photographs, and visual field was performed on all suitable patients. According to the inclusion/exclusion criteria (as summarized in the Table 1), 120 patients diagnosed with POAG and scheduled for anterior eye segment surgery (Watson’s trabeculectomy technique) gave informed consent to participate in this study and were finally enrolled and distributed into three groups: 1) smokers (S, n=40), 2) ex-smokers (ES, n=40) and 3) non-smokers (NS, n=40).

**Table 1:** Demographic characteristics of patients.

** Features**	**S mean (SD)**	**ES mean (SD)**	**NS mean (SD)**
Age (years)	71.5 (7.0)	70.1 (11.3)	72.3 (7.2)
BCVA (Snellen)	0.79 (0.10)	0.83 (0.12)	0.82 (0.12)
IOP (mmHg)	25.1 (3.2)	25.7 (3.4)	23.7 (2.8)

S=Smoker; ES=Ex-Smoker; NS=Non-Smoker; SD=standard deviation; BCVA=Best Corrected Visual Acuity; IOP=Intraocular Pressure

### Sampling procedures

Global standards for subject biosample workflow, transport, storage, and procedures were strictly followed. Aqueous humor samples were obtained from the eye at the onset of surgery by aspiration throughout corneal paracentesis, using a 27-gauge needle under an operating microscope (Zeiss). Samples were collected in cryotubes. Once the aqueous humor was drawn, the cryotube was labeled with patient data, and deposited into the freezer at −20 °C for 1 h. Then, the cryotubes were immediately transported to the laboratory to be frozen at −85 °C and to be logged in until processing. Blood samples were extracted from the antecubital vein at baseline. Then, plasma samples were obtained by centrifugation at 1,000 rpm and −4 °C for 10 min, collected in cryotubes and frozen at −85 °C.

### Analytical procedures

The aqueous humor (AH)  and plasma samples were processed for biochemical and western blot assays as follows.

#### Interleukin-6 level determination

This was performed by the Interleukin-6 Human EIA kit from Cayman Chemical (Cayman Chemical Co., Ann Arbor, MI), which is based on a double-antibody “sandwich” technique. An IL-6 molecule is binding for two antibodies: 1) monoclonal antibody specific for IL-6 and 2) acetylcholinesterase:Fab’ conjugate. The enzymatic activity of acetylcholinesterase is measured at 412 nm. The concentration of the IL-6 is proportional to the amount of bound conjugate.

#### Total protein concentration assays

This determination was made as a prelude to the western blot technique, to load the same amount of protein for each sample. The Bicinchoninic Acid Protein Assay Kit (Sigma-Aldrich, Bellefonte, PA) was used. In this assay, a Cu^2+^-protein complex is formed under alkaline conditions. Then, Cu^2+^ is reduced to Cu^1+^. Finally, the bicinchoninic acid (BCA) forms a purple-blue complex with Cu^1+^, and absorbance at 560 nm was measured by means of a spectrophotometer.

#### Western blot

Samples were prepared by western blot assays:

- Gel electrophoresis: 30 µg protein from each sample was loaded on gel (NuPAGE 4%–12% Bis-Tris Gel, Invitrogen, Philadelphia, PA). A molecular weight marker was also loaded (NuPAGE Magicmark XP Western Standard, Invitrogen). The electrophoresis was performed using a PowerEase 500 Power Supply (Invitrogen) at 150V for 90 min.
- Transfer to nitrocellulose membrane of 0.45 µm pore size (Invitrogen) was performed in the PowerEase 500 Power Supply at 30V for 60 min.
- Immunoblotting: A polyclonal antibody (PARP H-250, Santa Cruz Biotechnology Inc., Santa Cruz, CA) and Cleaved Caspase-3 (Asp175, Cell Signaling Technology, Danvers, MA) was used for determining PARP-1 and caspase-3 expression. Immunoblotting was performed by using a Invitrogen kit (WesternBreeze® Chromogenic Kit–Anti-Rabbit).

Optical density of the respective bands was determined using the Scion Image Analysis System (Scion Corp., Frederick, MD). Data were analyzed with the SPSS program (version 15.0 for Windows from SPSS Inc., Chicago, IL). We used the one-way ANOVA to compare the quantitative variables between groups and the Student *t* test for comparing two means. Significance level was set at a p-value of 0.05 or less by a two-tailed test.

## Results

There were no significant differences in age, best corrected visual acuity, or IOP between groups (Table 1). First, we performed an ANOVA (ANOVA, Table 2). After that, we performed a bivariate statistical analysis by means of the Student *t *test (Table 3) to estimate differences between the two means.

**Table 2:** ANOVA results in the aqueous humor and plasma samples.

**Biomarkers**		**Aqueous humor**					**Plasma**				
		**Sum of squares**	**df**	**Mean square**	**F**	**S.**	**Sum of squares**	**df**	**Mean square**	**F**	**S.**
IL-6 (pg/ml)	BG	2065173	2	1032586.657	392.370	1.308×10^-10^	333574.0	2	166787.006	47.965	6.125×10^-16^
	WG	307904.7	117	2631.664			406842.6	117	3477.288		
	Total	2373078	119				740416.7	119			
PARP-1 (rdu)	BG	11084.596	2	5542.298	199.638	1.929×10^-38^	1053.545	2	526.772	20.209	2.892×10^-8^
	WG	3248.115	117	27.762			3049.755	117	26.066		
	Total	14332.712	119				4103.300	119			
CASP-3 (rdu)	BG	1908.805	2	954.403	34.871	1.322×10^-12^	1722.728	2	861.364	25.013	9.035×10^-10^
	WG	3202.260	117	27.370			4029.061	117	34.436		
	Total	5111.065	119				5751.789	119			

IL-6=Interleukin-6; PARP-1=Poly(ADP-Ribose)Polymerase 1; CASP-3=Caspase 3; df=degrees of freedom; F=F statistic; S=Significance; rdu=relative densitometric units; BG=Between Groups; WG=Within Groups

**Table 3:** Results of analytical determinations in aqueous humor and plasma samples.

**Samples**	**Molecules**	**S Mean (SD)**	**ES Mean (SD)**	**NS Mean (SD)**	**p-value 1**	**p-value 2**	**p-value 3**
Aqueous humor	Interleukin-6 (pg/ml)	743.6 (42.7)	503.0 (56.4)	438.9 (53.9)	1.471×10^-34^	1.658×10^-42^	1.517×10-^6^
	PARP-1 (rdu)	59.9 (5.9)	40.7 (5.1)	38.5 (4.9)	7.661×10^-26^	3.072×10^-29^	0.047
	CASP-3 (rdu)	48.6 (5.8)	41.3 (4.6)	39.2 (5.2)	2.828×10^-8^	8.318×10^-11^	0.061*
Plasma	Interleukin-6 (pg/ml)	874.5 (66.2)	752.8 (54.5)	776.3 (55.5)	1.174×10^-13^	3.413×10^-10^	0.060*
	PARP-1 (rdu)	78.8 (5.4)	76.08 (5.7)	71.6 (4.0)	0.032	2.562×10^-9^	1.2×10-^4^
	CASP-3 (rdu)	61.9 (4.6)	53.5 (7.5)	54.4 (5.1)	4.020×10^-8^	1.082×10^-9^	0.504*

S=Smoker; ES=Ex-Smoker; NS=Non-Smoker; SD=Standard Deviation; PARP-1=Poly (ADP-Ribose) Polymerase-1; CASP-3=Caspase-3; rdu=relative densitometric units; p-value 1=Smoker versus Ex-Smoker; p-value 2=Smoker versus Non-Smoker; p-value 3=Ex-Smoker versus Non-Smoker. The asterisk indicates not significant (p>0.05).

**Figure 1:**
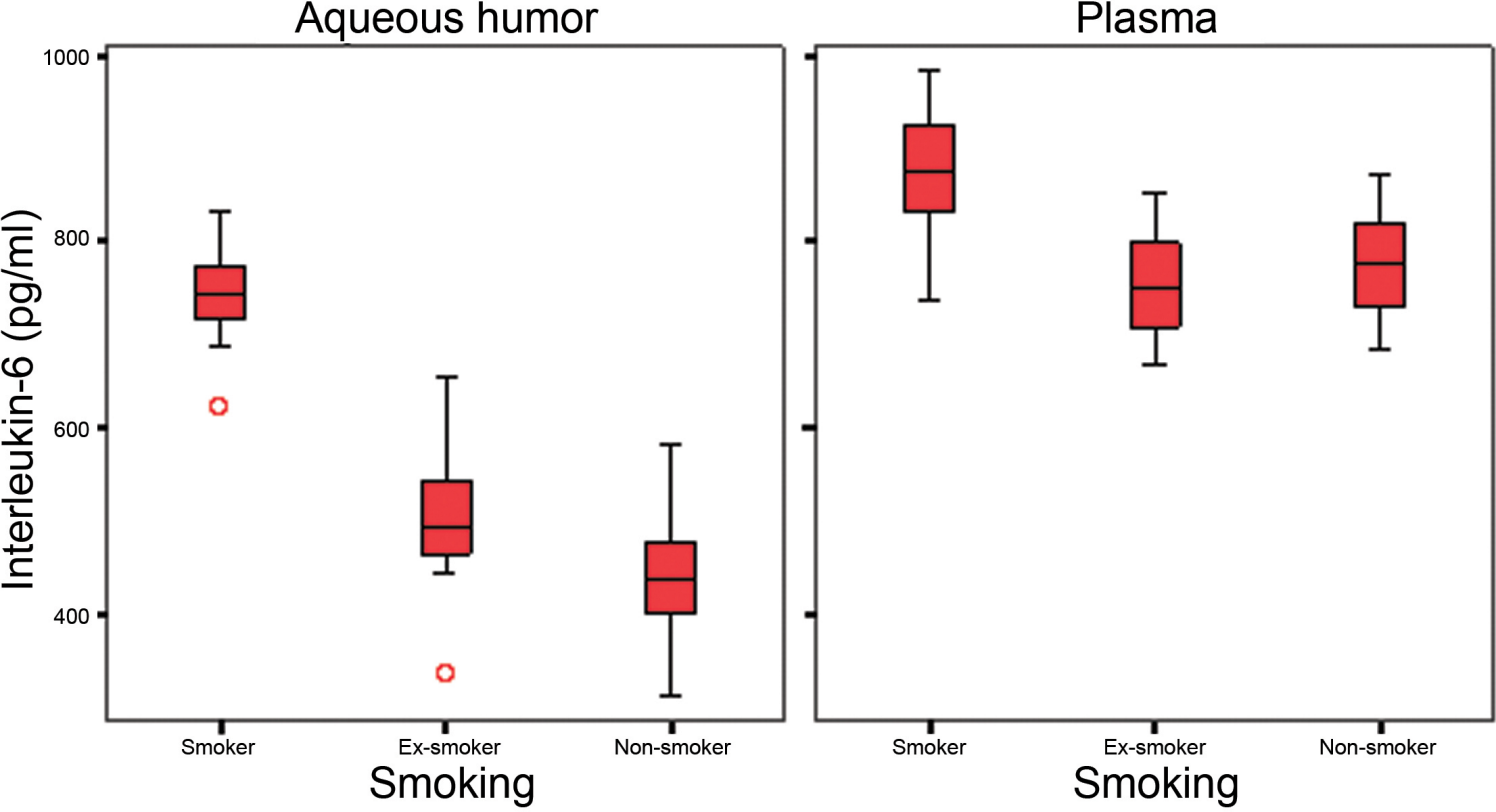
Levels of interleukin-6 in aqueous humor and plasma of the study groups.

**Figure 2:**
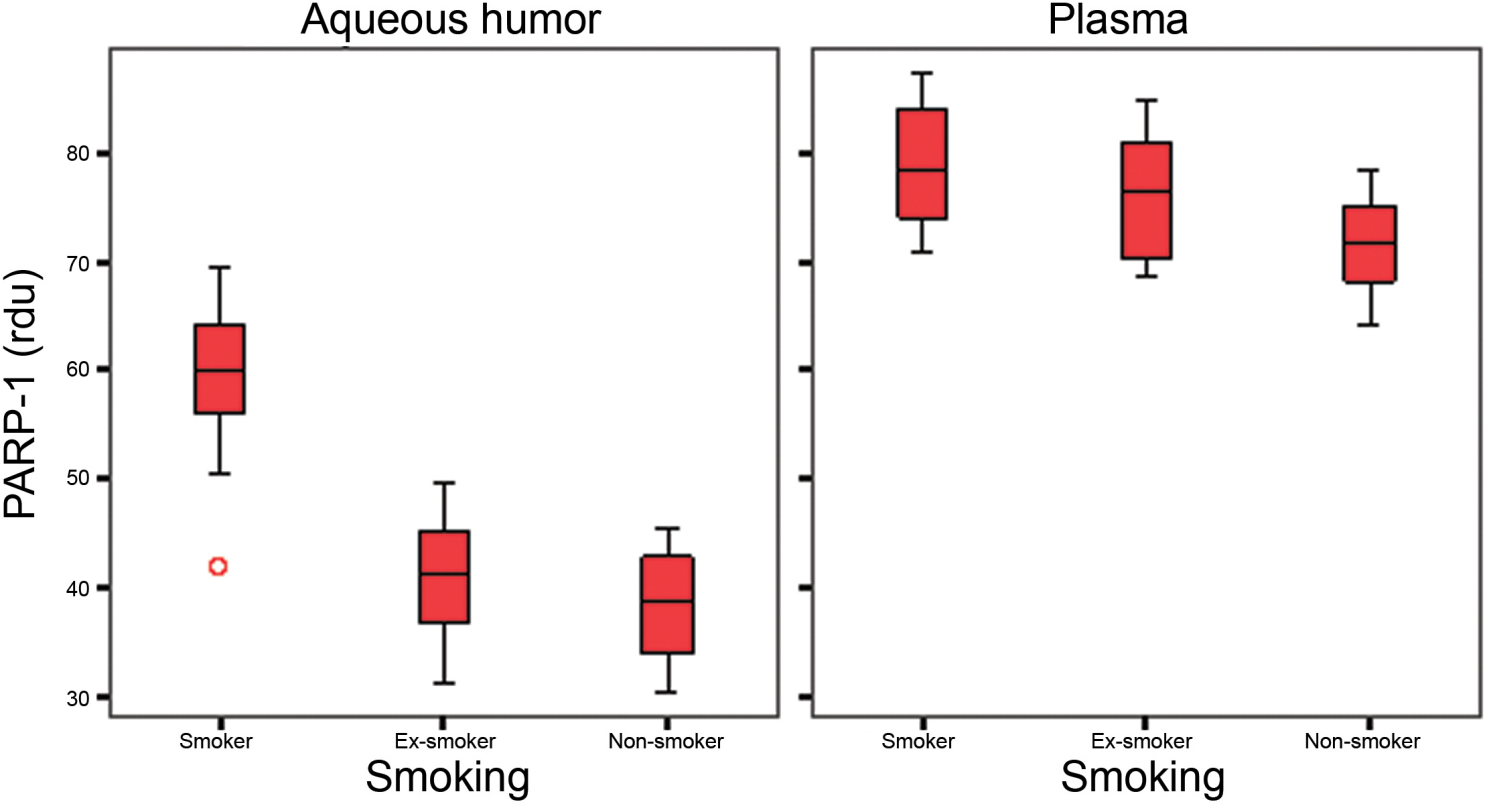
Poly (ADP-ribose) polymerase 1 levels (85 kDa band), measured as relative densitometric units (rdu), in both aqueous humor and plasma samples of the study groups.

**Figure 3:**
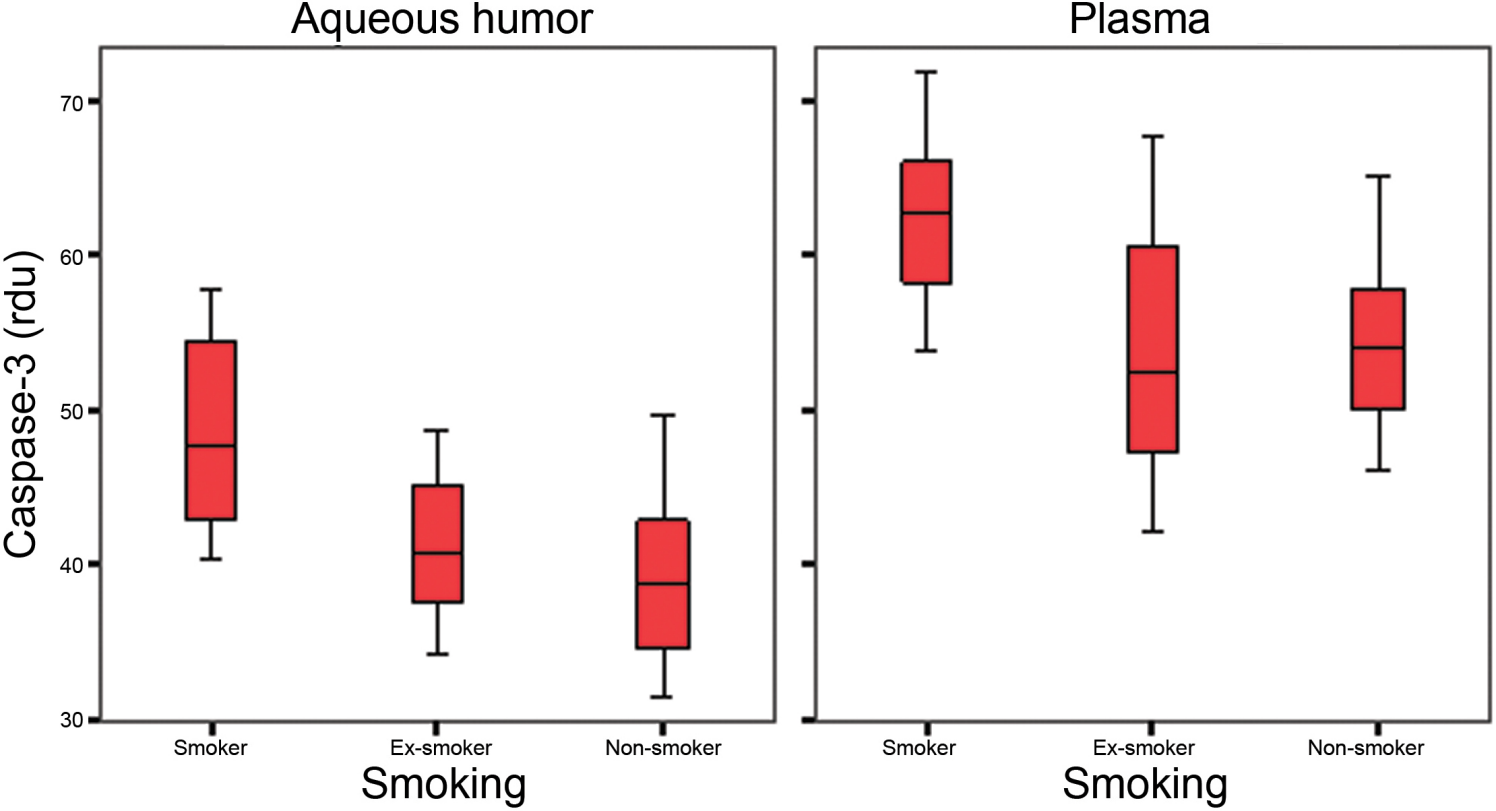
Caspase-3 levels (relative densitometric units – rdu) in aqueous humor and plasma samples of the study groups.

1. IL-6 levels were significantly different between groups for the aqueous humor and plasma. In the aqueous humor, IL-6 levels were significantly higher in the smoker group than in the ex-smoker group and also statistically higher in the ex-smoker group than in the non-smoker group. In the plasma samples, the IL-6 levels were statistically higher in the smoker group compared to the other two groups. However, the IL-6 levels were lower in the ex-smoker group than in the non-smoker group, but the difference was not statistically significant (Figure 1).
2. Data from the cleaved PARP-1 showed significant differences between the study groups for both aqueous humor and plasma. The levels of PARP-1 were significantly higher in the smoker group than in the ex-smoker group and also were statistically higher in the ex-smoker group than in the non-smoker group (Figure 2).
3. Caspase-3 levels were significantly different between our study groups for both aqueous humor and plasma samples. The levels of caspase-3 were statistically higher in smokers than in ex-smokers and non-smokers. In the aqueous humor, the caspase-3 levels were higher in the ex-smoker group than in the non-smoker group. However, the plasmatic caspase-3 levels were higher in the non-smoker group than in the ex-smoker group. For both aqueous humor and plasma samples, differences between the ex-smoker and non-smoker groups were not significant (Figure 3).

## Discussion

It is known that primary open-angle glaucoma is a major cause of irreversible blindness worldwide. Its cause remains unknown, so the study of the risk factors for this optic neuropathy is pivotal for managing the disease and avoiding the progression to blindness. Thus, we have studied the influence of smoking on the progression of this disease, taking into consideration that the eye is naturally exposed to the environment, which means that cigarette smoke can directly affect it.

Although the study was conducted in a population of women, there is no evidence that women have a higher risk of glaucoma than men. In any case, this neuropathy is a disease associated with age, and it has been shown that some hormonal factors influence POAG. Therefore, post-menopausal women present an increased risk of progression of this disease. The presence of more glaucomatous women than glaucomatous men in the general population may be because the prevalence of glaucomatous optic neuropathy increases with age and women have a higher life expectancy than men.

Grzybowski [40] has shown that smoking produces ischemia and oxidative stress and that smoking has a negative impact on POAG surgery. Cheng et al. [41] also found a strong association between smoking and glaucoma, suggesting that the damage from smoke is probably due to the presence of toxic substances that induce an increase in free radicals and a decrease in antioxidants.

In the present work we have investigated the role of smoking in the expression of inflammation (IL-6) and apoptosis (PARP-1, caspase-3) molecules in a gender-related fashion. Our results agree with those of other researchers and strongly suggest that cigarette smoking increases the expression of pro-apoptotic molecules, markers of cell death. Epidemiological [42] and experimental [43] studies have shown a link between the increased caspase-3 levels and glaucoma disease. Likewise, increased levels of cleaved PARP-1 [44] and increased rates of apoptosis have been previously described. In fact, these results are consistent with the association between apoptosic cell death and glaucoma found by other researchers [45,46].

Smoking is also associated with inflammation. Rodriguez [47] conducted a study that found a strong connection between the increase of C-reactive protein and smoking and established that this increase is directly related to the duration of smoking. In addition, it has been demonstrated that smoking increases oxidative stress [48] and that oxidative stress plays a role in acute and chronic inflammation [49]. Because of this, we have evaluated herein the interleukin-6 levels in the aqueous humor and plasma, and our results show that smoking influences the IL-6 levels in glaucomatous females. Rummenie et al. [50], in a study about cigarette smoke effects on tear and the ocular surface, observed that IL-6 concentrations increased significantly in tear 24 h after smoke exposure. Studying the progression of ARMD, Seddon et al. [51] also found a relationship between IL-6 levels and smoking.

Data presented in this work are consistent with the fact that habitual smoking increases the expression of inflammatory and apoptosis markers in glaucoma patients. Since it is known that RGC die by apoptosis in glaucoma, we theorized that the increase of apoptotic and inflammation markers in POAG due to smoking might contribute to the progression of the disease.

In this work we studied the changes produced in the expression of certain molecules by tobacco usage in a population of women, and we observed how the expression of the molecules studied increased with smoking. However, there are some factors that we have not considered in our study, such as passive smoking. Passive smoking is a universal problem, and all people (smokers, ex-smokers, or nonsmokers) may be exposed to tobacco smoke in public places, at home, or at work. However, smokers do not usually have a problem staying in smoky places, while nonsmokers tend to avoid them.

We are currently undertaking a project to attempt to evaluate whether smoking influences the expression of the same molecules in men with glaucoma. Subsequently, if the answer to this new study is positive, we will examine whether the influence of smoking on inflammatory and apoptotic processes is greater in women or in men.

In summary, further research is needed in the context of glaucoma biomarkers, the influence of exogenous risk factors (such as smoking habits), IOP, and the morphological and functional glaucoma test to recommend avoiding smoking, particularly in older women.
